# PLK1 blockade enhances therapeutic effects of radiation by inducing cell cycle arrest at the mitotic phase

**DOI:** 10.1038/srep15666

**Published:** 2015-10-27

**Authors:** Minoru Inoue, Michio Yoshimura, Minoru Kobayashi, Akiyo Morinibu, Satoshi Itasaka, Masahiro Hiraoka, Hiroshi Harada

**Affiliations:** 1 Department of Radiation Oncology and Image-applied Therapy, Kyoto University Graduate School of Medicine, 54 Shogoin Kawahara-cho, Sakyo-ku, Kyoto 606-8507, Japan; 2 Group of Radiation and Tumor Biology, Career-Path Promotion Unit for Young Life Scientists, Kyoto University, Yoshida Konoe-cho, Sakyo-ku, Kyoto 606-8501, Japan; 3 Precursory Research for Embryonic Science and Technology (PRESTO), Japan Science and Technology (JST), 4-1-8 Honcho, Kawaguchi, Saitama 332-0012, Japan; 4 Hakubi Center, Kyoto University, Yoshida-Honmachi, Sakyo-ku, Kyoto 606-8501, Japan

## Abstract

The cytotoxicity of ionizing radiation depends on the cell cycle phase; therefore, its pharmacological manipulation, especially the induction of cell cycle arrest at the radiosensitive mitotic-phase (M-phase), has been attempted for effective radiation therapy. Polo-like kinase 1 (PLK1) is a serine/threonine kinase that functions in mitotic progression, and is now recognized as a potential target for radiosensitization. We herein investigated whether PLK1 blockade enhanced the cytotoxic effects of radiation by modulating cell cycle phases of cancer cells using the novel small molecule inhibitor of PLK1, TAK-960. The TAK-960 treatment exhibited radiosensitizing effects *in vitro*, especially when it increased the proportion of M-phase cells. TAK-960 did not sensitize cancer cells to radiation when an insufficient amount of time was provided to induce mitotic arrest. The overexpression of a PLK1 mutant, PLK1-R136G&T210D, which was confirmed to cancel the TAK-960-mediated increase in the proportion of mitotic cells, abrogated the radiosensitizing effects of TAK-960. A tumor growth delay assay also demonstrated that the radiosensitizing effects of TAK-960 depended on an increase in the proportion of M-phase cells. These results provide a rational basis for targeting PLK1 for radiosensitization when considering the therapeutic time window for M-phase arrest as the best timing for radiation treatments.

Cancer patients often develop the local recurrence and distant metastases of malignant tumors following radiation therapy. Escalations in the radiation dose have been used to overcome these problems; however, the dose that cancer patients can receive is limited due to side effects in the surrounding normal tissues. It is widely accepted that the combination of chemotherapeutic agents with radiation therapy, which is called chemo-radiotherapy, is a rational strategy for sensitizing cancer cells to radiation therapy and compensating for lower radiation doses.

Another advantage of administering chemotherapeutic agents in combination with radiation is the ability to compensate for the limitation of radiation therapy and consequently increase the rate of complete remission by inducing damage in radioresistant cancer cells. Although various factors have been reported to influence the radioresistance of cancer cells, one of the most important has been identified as the cell cycle status. M-phase is recognized as a radiosensitive phase^1^ because DNA double-strand break (DSB) repair is inactivated in mitotic cells through the phosphorylation of the E3 ubiquitin ligase, RNF8, and the tumor suppressor p53 binding protein 1, 53BP1 by mitotic kinases^2^. Therefore, genes that are responsible for the progression of this cell cycle phase are considered to be potential targets for enhancing the therapeutic effects of radiation.

Polo-like kinase 1 (PLK1) is a serine/threonine kinase that plays important roles throughout the progression of the mitotic phase, including centrosome maturation, mitotic entry, chromosome segregation, and cytokinesis^3^. PLK1 is activated through the phosphorylation of its threonine 210 residue (T_210_) by Aurora-A in a Bora-dependent manner^4^,^5^. The active form promotes entry into the mitotic phase, *e.g.* by phosphorylating and activating Cdc25C^6^,^7^. PLK1 was previously shown to be overexpressed in various types of cancers and is known to be responsible for the aberrant proliferation of cancer cells. Another important function of PLK1 is to control DNA damage responses through the regulation of p53 binding protein 1 (53BP1) and claspin activities^8^,^9^. For example, PLK1 was reported to directly bind 53BP1 during mitosis and inactivate the DNA damage checkpoint^9^. Moreover, the intratumoral expression levels of PLK1 have been correlated with the poor prognoses of patients with various types of cancers^10^,^11^,^12^,^13^,^14^,^15^,^16^,^17^,^18^. These findings justify both the development of PLK1 inhibitors as anti-cancer agents and their application to chemoradiotherapy.

In the present study, we assessed the importance of PLK1 blockade-mediated mitotic arrest in sensitizing cancer cells to radiation therapy by using the novel small molecule inhibitor of PLK1, TAK-960^19^,^20^. We herein demonstrated, for the first time, the radiosensitizing effects of TAK-960 and provided direct evidence for the involvement of mitotic arrest in the radiosensitizing effects of PLK1 blockade using the mutant type of PLK1 mimicking T210 phosphorylation.

## Results

### Optimal TAK-960 treatment for mitotic arrest *in vitro*

**Figure 1:**
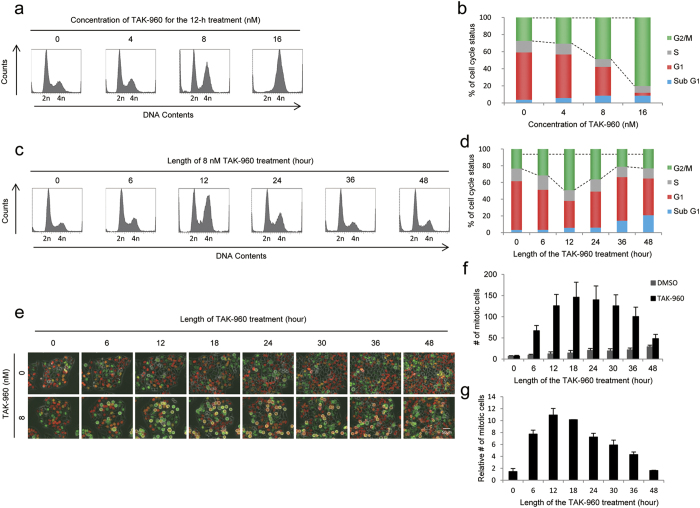
Optimal TAK-960 treatment for mitotic arrest *in vitro*. (**a**–**d**) After being treated with the indicated concentrations of TAK-960 for the indicated durations, HeLa cell suspensions were subjected to flow cytometric analyses of cell cycle phases. (**a**,**c**) Representative data are shown. (**b**,**d**) The proportions of cells in each phase were quantified based on the data in (**a,b**), respectively. Results are the mean. *n* = 3. (**e**–**g**) HeLa-S FUCCI cells were treated with 0 or 8 nM TAK-960 and subjected to time-lapse imaging. (**e**) Representative images are shown. Bar = 50 μm. (**f**) The number of cells in the mitotic phase per one field was quantified. Results are the mean ± s.d. *n* = 15 (5 fields in 3 independent experiments). (**g**) The relative number of mitotic cells with the TAK-960 treatment to that with the DMSO treatment at each time point was quantified based on the data in Fig. 1f. Results are the mean ± s.d. *n* = 15 (5 fields in 3 independent experiments).

Accumulating evidence in the research field of radiation biology has demonstrated that the radiosensitivity of cells depends on the phases of the cell cycle; cells are relatively radioresistant in the late S-phase, but are radiosensitive in the mitotic phase (M-phase). This fundamental principle prompted us to hypothesize that the small molecule inhibitor of PLK1, TAK-960, which has been reported to cause the mitotic arrest of cancer cells^19^, may enhance the cytotoxic effects of radiation. In order to test this possibility, we first determined the optimal conditions of the TAK-960 treatment for mitotic arrest without apparent cytotoxic effects *in vitro*. We treated human cervical cancer cells, HeLa, with various concentrations of TAK-960 and performed cell cycle analyses using flow cytometry with propidium iodide. Among the various concentrations of TAK-960 examined, 16 nM resulted in the most pronounced cell cycle arrest at the G_2_/M phase (Fig. 1a,b), but had a significant cytotoxic effect in the clonogenic survival assay (Supplementary Fig. S1 online). On the other hand, lower concentrations of TAK-960, such as 8 nM, led to G_2_/M cell cycle arrest without significant cytotoxicity. We then treated HeLa cells with 8 nM TAK-960 for various periods to optimize the duration of the treatment (Fig. 1c,d). Flow cytometric analyses revealed that the proportion of cells in the G_2_/M phase was maximized by the TAK-960 treatment for 12 hours.

In order to monitor the influence of the TAK-960 treatment on the cell cycle status in real-time, we employed HeLa-S FUCCI cells^21^, which preferentially emit orange (monomeric Kusabira-Orange2: mKO2) and green fluorescence (monomeric Azami-Green1: mAG1) in the G_1_-phase and S/G_2_/M-phase, respectively. Time-lapse imaging confirmed that cells alternatively exhibited orange and green fluorescence in the absence of the TAK-960 treatment, and the number of round-shaped cells with green fluorescence, which represent mitotic cells, slightly increased, corresponding to proper cell cycle progression (Fig. 1e,f; Supplementary Video S1 online). Upon exposure to 8 nM TAK-960, the number of mitotic cells markedly increased and peaked 12–18 hours after the initiation of the TAK-960 treatment, which was consistent with the results of the flow cytometric analyses (*compare* Fig. 1c,d with 1e–g; Supplementary Video S2 online). Taken together, these results indicated that the optimal condition for the most pronounced cell cycle arrest at the mitotic phase was 8 nM TAK-960 for 12 hours.

### Radiosensitizing effects of TAK-960 *in vitro*

**Figure 2:**
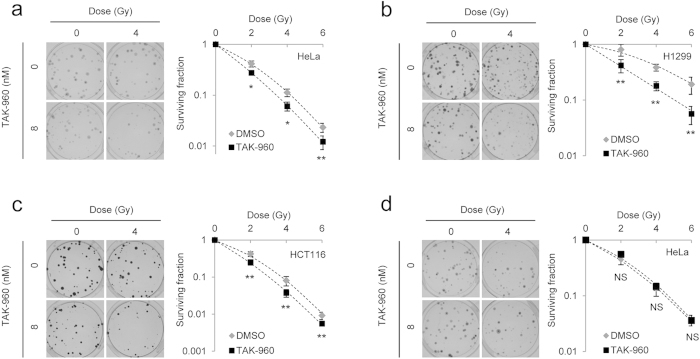
Radiosensitizing effects of TAK-960 *in vitro*. (**a**–**c**) A clonogenic survival assay was performed using HeLa (**a**), H1299 (**b**), and HCT116 (**c**) treated with 0 or 8 nM TAK-960 for 12 h and with the indicated dose of X-irradiation. Representative images (left in each panel) and the surviving fraction (right in each panel) are shown. Results are the mean ± s.d. *n* = 3. *P < 0.05, **P < 0.01. (**d**) A clonogenic survival assay was performed using HeLa cells treated with the indicated dose of X-irradiation just after the addition of 0 or 8 nM TAK-960 to the culture medium. Representative images (left) and the surviving fraction (right) are shown. Results are the mean ± s.d. *n* = 3. NS = not significant.

We performed clonogenic cell survival assays *in vitro* to determine whether the optimized TAK-960 treatment actually enhanced the cytotoxic effects of X-irradiation. Not only HeLa cells, but also human non-small cell lung cancer cells, H1299, and human colon cancer cells, HCT116, were subjected to the same TAK-960 treatment (8 nM TAK for 12 hours) before X-irradiation because we confirmed that the TAK-960 treatment clearly induced the mitotic arrest of H1299 and HCT116 cells (Fig. 2a–c, Supplementary Fig. S2 online). Without the radiation treatment, the number of surviving colonies was similar between the control (0 nM) and TAK-960 treatment (8 nM) groups, which is consistent with the observations in Supplementary Fig. S1 online. On the other hand, the TAK-960 treatment significantly decreased clonogenic survival after X-irradiation. The dose of radiation required to reduce the number of surviving colonies by 90% (D_10_ value) was significantly decreased by the TAK-960 treatment from 4.2 ± 0.2 to 3.4 ± 0.3 Gy in HeLa, from 7.5 ± 0.6 Gy to 5.0 ± 0.6 Gy in H1299, and from 3.7 ± 0.3 Gy to 3.0 ± 0.2 Gy in HCT116 cells (Table 1). The radiation enhancement ratio of the TAK-960 treatment based on the D_10_ value in HeLa cells was 1.24 ± 0.4, which was similar to that of another PLK1 inhibitor, BI2536 (1.26 ± 0.3; Supplementary Fig. S3 online).

Since TAK-960 was removed from the culture medium just before irradiation in the clonogenic cell survival assays, these results indicated that a cellular response to the TAK-960 pretreatment, possibly mitotic arrest, was required for its radiosensitizing effects. Consistent with this result, the concomitant administration of TAK-960 and X-irradiation, in which cells were exposed to the drug just before irradiation and did not have sufficient time to induce any cellular responses including cell cycle arrest, did not exhibit any radiosensitization (Fig. 2d).

### Importance of mitotic arrest in the radiosensitizing effects of TAK-960

**Figure 3:**
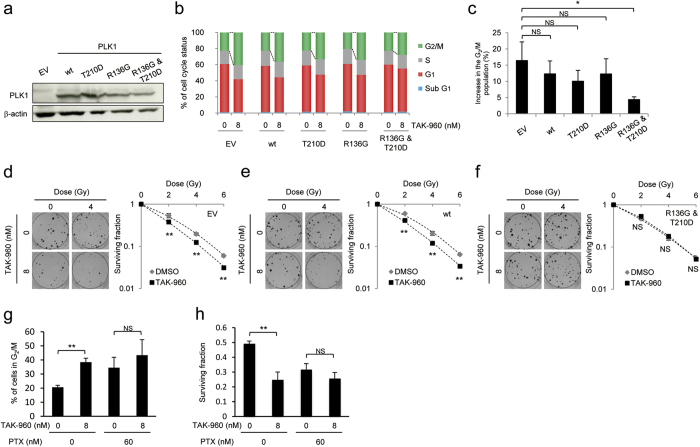
Importance of mitotic arrest for the radiosensitizing effects of TAK-960. (**a**–**f**) HeLa cells were transiently transfected with either pcDNA3.1/myc-His (empty vector: EV), pcDNA3.1/PLK1 (wild type: wt), pcDNA3.1/PLK1-R136G (R136G), pcDNA3.1/PLK1-T210D (T210D), or pcDNA3.1/PLK1-R136G&T210D (R136G&T210D) and precultured for 48 h. (**a**) Cell lysates were directly subjected to Western blotting for PLK-1 and β-actin. (**b**) Cells were then treated with 0 or 8 nM TAK-960 for 12 h and subjected to flow cytometric analyses of cell cycle phases. The proportions of cells in each phase were quantified. Results are the mean ± s.d. *n* = 3. *P < 0.05, NS = not significant. (**c**) Percentage increases in cells in the G_2_/M phase by the TAK-960 treatment were quantified based on the data in (**b**). *P < 0.05, NS = not significant. (**d**–**f**) Cells were treated with 0 or 8 nM TAK-960 for 12 h and with the indicated dose of X-irradiation, and then subjected to clonogenic cell survival assays. Representative images (left in each panel) and cell survival curves (right in each panel) for EV (**d**), wt (**e**), and R136G&T210D (**e**) are shown. Results are the mean ± s.d. *n* = 3. **P < 0.01, NS = not significant. Original gel images of data (**a**) are presented in Supplementary Fig. S6 online. (**g**,**h**) HeLa cells were treated with or without Paclitaxel (60 nM) for 24 h and subsequently with or without TAK-960 (8 nM) for 12 h, and were subjected to flow cytometer-based cell cycle analyses (**g**) or to the clonogenic cell survival assay after 0 or 2 Gy X-irradiation. Results are the mean ± s.d. *n* = 3. **P < 0.01, NS = not significant.

In order to directly examine whether the radiosensitizing effects of the TAK-960 treatment were dependent on its PLK1-inhibiting activity and resultant mitotic arrest, we employed mutant forms of PLK1, which were previously reported to be less sensitive to PLK1 inhibitors, and thus, promoted M-phase progression even in the presence of the inhibitor. We constructed expression vectors for the wild-type (wt) and three mutant forms of PLK1: a phosphorylation-mimicking mutant, PLK1-T210D^22^,^23^, a mutant less sensitive to PLK1 inhibitors, PLK1-R136G^24^, and a mutant with both mutations, PLK1-R136G&T210D (Fig. 3a). Flow cytometry-based cell cycle analyses using propidium iodide revealed that the forced expression of these mutants had no effect on the cell cycle distribution of HeLa cells in the absence of the TAK-960 treatment (Fig. 3b,c). In contrast, the TAK-960-mediated increase in the proportion of cells in the G_2_/M phase was significantly suppressed by the overexpression of PLK1-R136G&T210D, but not that of PLK1-T210D or PLK1-R136G in our experimental setting (Fig. 3b,c). Based on these results, we employed the expression vector for PLK1-R136G&T210D and directly tested the importance of mitotic arrest in the radiosensitizing effects of the PLK1 inhibitor, TAK-960 (Fig. 3d–f). Clonogenic cell survival assays demonstrated that the radiosensitizing effects of TAK-960 were almost completely abolished by the introduction of PLK1-R136G&T210D (*compare* Fig. 3f with Fig. 3d,e).

We employed paclitaxel (PTX), which is known to induce cell cycle arrest at the mitotic phase as an inhibitor of microtubular depolymerization^25^ to further test the importance of mitotic arrest in TAK-960-mediated radiosensitization. The clonogenic cell survival assay demonstrated that the relative radiosensitizing effects of TAK-960 were significantly decreased after the cell cycle was synchronized at the mitotic phase by paclitaxel (Fig. 3g,h). These results clearly showed that TAK-960 enhanced the cytotoxic effects of radiation through its own PLK1-inhibiting activity and consequent cell cycle arrest at the radiosensitive mitotic phase.

### Optimal TAK-960 treatment for mitotic arrest *in vivo*

**Figure 4:**
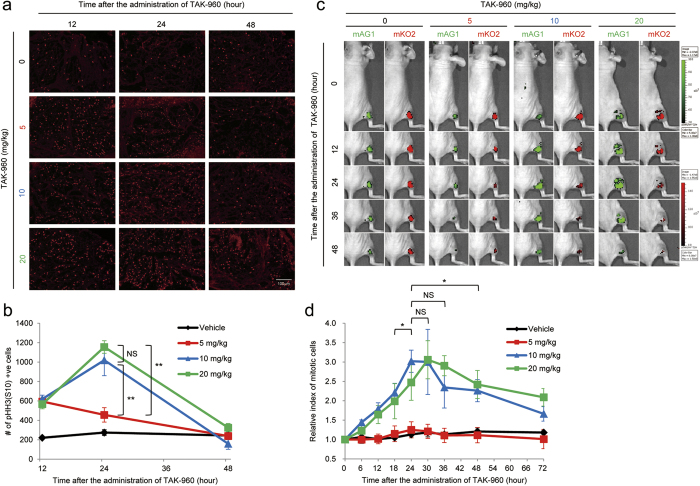
Optimal TAK-960 treatment for mitotic arrest *in vivo*. (**a**,**b**) Twelve, 24, and 48 hours after mice bearing a subcutaneous HeLa tumor xenograft were administered with the indicated dose of TAK-960, tumor xenografts were subjected to immunohistochemical analyses using an antibody against phosphorylated histone H3 (Ser10) (pHH3(S10)). (**a**) Representative images are shown. Bar = 100 μm. (**b**) The number of pHH3(S10)-positive cells are quantified based on the data in Fig. 4a. *n* = 15 (5 fields in 3 independent tumors). **P < 0.01, NS = not significant. (**c**,**d**) Mice bearing a subcutaneous HeLa-S FUCCI tumor xenograft were administered with the indicated dose of TAK-960, and subjected to optical image analyses with the IVIS-SPECTRUM imaging system for mAG1 (green) and mKO2 (red). (**c**) Representative figures are shown. (**d**) The index of mitotic cells at each time point was firstly calculated as the relative intensity of green fluorescence (mAG1) to that of red fluorescence (mKO2) based on the data in Fig. 4c. The relative index of mitotic cells was calculated as the ratio of the index at each time point to that at the initial time point. *n* = 3. *P < 0.05. NS = not significant.

Prior to evaluating the radiosensitizing effects of TAK-960 *in vivo*, we determined the optimal TAK-960 treatment to maximize the amount of cells in mitotic arrest without influencing xenografted tumor growth *in vivo*. We first analyzed changes in the number of mitotic cells after the administration of TAK-960 by immunohistochemistry. Mice bearing a subcutaneous HeLa tumor xenograft were administered with various doses of TAK-960 and subjected to immunohistochemical analyses using an antibody against a marker of mitotic cells, phosphorylated histone H3 (Ser10) (pHH3(S10)). The maximal number of pHH3(S10)-positive cells was observed 24 hours after the administration of 10 or 20 mg/kg TAK-960 (Fig. 4a,b). In order to confirm this result, we conducted an optical *in vivo* imaging experiment using the HeLa-S FUCCI tumor xenograft model. The use of green fluorescence as an indicator of mitotic cells was justified by immunocytochemical data in which the TAK-960 treatment significantly increased the proportion of cells that were round in shape, emitted green fluorescence, and were stained with the marker of mitotic cells, pHH3(S10) (Supplementary Fig. S4 online; Supplementary Table S1 online). Subcutaneous HeLa-S FUCCI tumor xenografts were injected with the indicated dose of TAK-960, and green and orange fluorescence from mAG1 and mKO2, respectively, was detected by the IVIS-SPECTRUM imaging system in real-time (Fig. 4c). In order to exclude the possibility that the intensity of fluorescence was influenced by tumor growth (increases in tumor volume over time), we calculated the ratio of the intensity of green fluorescence (mAG1) to that of red fluorescence (mKO2) as an index of mitotic cells (Fig. 4d). The mitotic index was significantly increased and peaked 24–36 hours after the administration of 10 or 20 mg/kg TAK-960, which was consistent with the immunohistochemical data for pHH3(S10) (Fig. 4d). We then assessed the influence of the various doses of the TAK-960 treatment on the growth of HeLa tumor xenografts. The tumor growth delay assay revealed that 10 mg/kg was the maximum dose that did not influence tumor growth and did not reduce the body weights of mice (Supplementary Fig. S5 online; Supplementary Table S2 online). Collectively, the optimal conditions that caused significant cell cycle arrest at the M-phase with minimum effects on tumor growth *in vivo* for subsequent *in vivo* experiments were determined to be 24 hours after the administration of 10 mg/kg TAK-960.

### Radiosensitizing effects of TAK-960 *in vivo*

**Figure 5:**
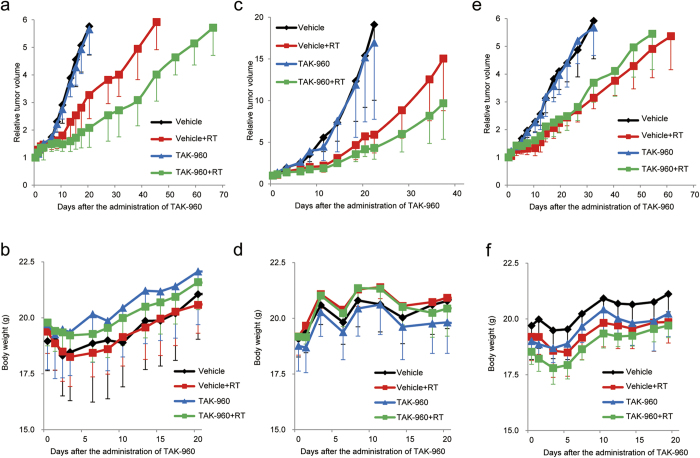
Radiosensitizing effects of TAK-960 *in vivo*. Twenty-four hours after (**a**–**d**) or just after (**e**,**f**) the oral administration of 0 (black and red lines) or 10 mg/kg (blue and green lines) TAK-960, mice bearing a HeLa (**a**,**b**,**e**,**f**) or H1299 (**c**,**d**) tumor xenograft were subjected to local irradiation at a dose of 0 (black and blue lines) or 10 (red and green lines) Gy. (**a**,**c**,**e**) Relative tumor volumes are calculated as the ratio of the tumor volume on each day to the corresponding volume on day 0. Results are the means ± s.d. *n* = 8. (An animal in the TAK960 + RT group in c and d died on day 1 for undefined reasons). (**b**,**d**,**f**) Body weights of mice in the experiment shown in Fig. 5a,c,e, respectively.

In order to examine whether the TAK-960 treatment in the optimized treatment regimen actually enhanced the therapeutic effects of radiation *in vivo*, we performed tumor growth delay assays using the subcutaneous HeLa tumor xenograft model (Fig. 5a; Table 2). Tumor xenografts were locally irradiated at a dose of 0 or 10 Gy 24 hours after the oral administration of 0 or 10 mg/kg TAK-960. We intentionally selected a relatively low dose of TAK-960 (10 mg/kg), which was confirmed to be sufficient for mitotic arrest with minimal effects on the tumor growth rate (Fig. 4; Supplementary Fig. S5 online); therefore, tumor growth after the TAK-960 treatment alone was not significantly delayed more that with the sham treatment. Tumor growth doubling time (TGDT) after the TAK-960 treatment alone was 8.3 ± 1.6 days and was not significantly longer than that after the sham treatment (9.1 ± 1.7 days; *P* = 0.33). Conversely, the TAK-960 treatment significantly suppressed tumor growth when combined with IR. TGDT after the combination of radiation therapy with the TAK-960 treatment was 27.4 ± 10.0 days, and was approximately 2.0-fold longer than that after radiation therapy (14.0 ± 5.1 days; *P* < 0.05). The same radiosensitizing effects of TAK-960 were confirmed in another tumor xenograft model with human non-small cell lung cancer cells, H1299 (Fig. 5c; Table 2). This combination did not cause any obvious side effects (Fig. 5b,d). Taken all together, these results demonstrated that the TAK-960 treatment exhibited radiosensitizing effects in the optimized schedule. In order to further examine the importance of mitotic arrest in the radiosensitizing effects of TAK-960 *in vivo*, we intentionally irradiated subcutaneous HeLa tumor xenografts just after the administration of TAK-960, and TAK-960 was not allowed to induce mitotic arrest. A delay in tumor growth was not observed even after the combination of TAK-960 with radiation (Fig. 5e,f; Table 3), which supported our conclusion that the radiosensitizing effects of TAK-960 *in vivo* may be attributed to mitotic arrest.

## Discussion

In the present study, we determined whether the inhibition of PLK1 enhanced the cytotoxic effects of radiation by modulating the cell cycle phase of cancer cells. The novel small molecule inhibitor of PLK1, TAK-960, was confirmed to exhibit expected radiosensitizing effects when radiation was administered because TAK-960 increased the proportion of M-phase cells. This radiosensitizing function was almost completely abrogated by the forced expression of PLK1-R136G&T210D, which functions in mitotic progression even in the presence of TAK-960, further strengthening the importance of mitotic arrest for radiosensitization. Although previous studies indirectly suggested the relevance of mitotic arrest to the radiosensitizing effects of PLK1 inhibitors^26^,^27^,^28^,^29^,^30^,^31^, this is the first study to clearly demonstrate the direct involvement of mitotic arrest.

Although the present study validated the importance of mitotic arrest, we have to consider other possible mechanisms underlying the radiosensitizing effects of TAK-960. For example, Liu *et al.* identified PLK1 as a rational target for cancer therapy by demonstrating that PLK1 blockade induced apoptosis in cancer cells because of the inhibited ability of PLK1 to suppress the pro-apoptotic function of p53^32^. This finding indicated that the radiosensitizing effects of TAK-960 were due to p53-dependent apoptosis. Moreover, as described above, accumulating evidence has demonstrated that PLK1 functions in DNA damage responses as well through the modulation of 53BP1 and claspin activities^8^,^9^, and, therefore, suggests the importance of such a function in the radiosensitizing activity of TAK-960. Further studies are needed in order to more fully understand the molecular mechanisms underlying TAK-960-mediated radiosensitization.

TAK-960 as well as other PLK1 inhibitors, such as BI2536, have been confirmed to generate polyploidy cells^19^. However, polyploidy cells were only observed when cells were treated with a higher dose of TAK-960, *e.g.* 60 nM or higher. Therefore, we assumed that the radiosensitizing effects of TAK-960 did not result from the generation of polyploid cells, at least in our experimental setting (TAK-960 conc. = 8nM).

Our flow cytometry-based cell cycle analyses using a series of PLK1 mutants demonstrated that TAK-960-mediated increases in the number of mitotic cells were significantly suppressed by the overexpression of PLK1-R136G&T210D, but not by that of PLK1-R136G or PLK1-T210D. In contrast, a previous study reported that a R136G mutation was adequate to suppress mitotic arrest by another PLK1 inhibitor, BI2536^24^; however, this inhibitor was predicted to inhibit PLK1 through the same mechanism of action as TAK-960 because of their structural similarities. This discrepancy may have resulted from differences in the experimental settings. We performed every *in vitro* experiment without synchronizing cell cycles and simply introduced PLK1-R136G&T210D, which has the phosphorylation-mimicking mutation, T210D, in addition to R136G. On the other hand, in the previous study, PLK1-R136G-overexpressing cells were synchronized at the mitotic phase, in which the T210 residue is known to be phosphorylated. Therefore, our result is not only consistent with the previous finding, but, when taken together, indicates the importance of T210 phosphorylation in addition to the R136G mutation in the resistance of cells to PLK1 inhibitors.

The total dose of radiation is commonly fractionated in recent radiation therapies for cancer patients because fractionated radiation therapy has several advantages over single-dose radiation therapy^33^. Considering the cell cycle of cancer cells, a small dose of radiation in the first round is assumed to preferentially kill radiosensitive G_2_- and M-phase cells, but fails to damage radioresistant S-phase cells. Since the cell cycle of the surviving cells will be redistributed to subsequent phases, including the radiosensitive phases, a second round of radiation at the correct time, such as 24 hours after the first treatment, is expected to kill the fraction of surviving cells. Based on this notion, known as the redistribution of the cell cycle, PLK1 inhibitors including TAK-960 also have the ability to redistribute the cell cycle of the surviving cells to the radiosensitive M-phase with fractionated radiation therapy, resulting in radiosensitization. Moreover, PLK1 inhibitors may be particularly useful in hypo-fractionated radiation therapy, in which fractionated radiation is given at an interval of every few days, and, therefore, cannot theoretically take advantage of the redistribution of cell cycles for the enhancement of its therapeutic effect.

Radiation oncologists have recently obtained powerful tools, such as intensity-modulated radiation therapy (IMRT)^34^ and stereotactic radiation therapy (SRT)^35^, which enable a higher dose of radiation to be accurately and specifically delivered to a malignant tumor. However, even the most innovative strategies have failed to achieve complete remission; tumor recurrence, distant metastasis, and/or side effects in normal tissues have often been reported following radiation therapy. Based on our result in which TAK-960 enhanced the therapeutic effects of radiation with minimal side effects, TAK-960 is expected to contribute to achieving the same or stronger therapeutic effects even when combined with a reduced dose of radiation. A decrease in the radiation dose may also reduce the incidence of side effects associated with radiation therapy.

## Methods

### Cell culture and reagents

The human cervical epithelial adenocarcinoma cell line (HeLa) and human lung carcinoma cell line (H1299) were purchased from the American Type Culture Collection (Manassas, VA, USA). The human colon cancer cell line (HCT116) and HeLa-S FUCCI cell line were kindly provided by Prof. Bert Vogelstein (The Johns Hopkins University, Baltimore, MD) and RIKEN BRC, respectively. Cells were cultured with 10% FBS-containing Dulbecco’s modified Eagle’s medium (DMEM) in a well-humidified incubator with 5% CO_2_ and 95% air at 37 °C. TAK-960 was dissolved in DMSO and 0.5% (w/v) hydroxylpropyl methylcellulose solution for *in vitro* and *in vivo* experiments, respectively.

### Plasmid DNA

In order to construct the plasmid pcDNA3.1/PLK1, the coding sequence of the PLK1 gene was amplified by PCR from the HeLa cDNA library using the following primers: (5′-AAAAGCTTACCATGAGTGCTGCAGTGACTG-3′ and 5′-AGCGGCCGCTTAGGAGGCCTTGAGACGG-3′), and inserted between the HindIII-NotI sites of pcDNA3.1/myc-His C (Invitrogen Corp., Carlsbad, CA, USA). Regarding construction of the plasmids pcDNA3.1/PLK1-R136G, pcDNA3.1/PLK1-T210D, and pcDNADNA3.1-PLK1-R136G&T210D, PCR-based site-directed mutagenesis was performed using the following primer sets: (5′-TGCCGCCGGGGGTCTCTCCTGGAGCTGCAC-3′ and 5′-CAGGAGAGACCCCCGGCGGCAGAGCTCC-3′ for R136G, and 5′-AGGAAGAAGGACCTGTGTGGGACTCCTAAT-3′ and 5′-CCCACACAGGTCCTTCTTCCTCTCCCCGTC-3′ for T210D), and the resultant DNA fragments encoding PLK1-R136G, PLK1-T210D, and PLK1-R136G&T210D were inserted between the HindIII-BamHI sites of pcDNA3.1/PLK1.

### Flow cytometric analysis, Western blotting, and time-lapse imaging

HeLa cells were seeded in 6-cm dishes for flow cytometric analyses (2 × 10^5^ cells/dish) or in 6-well plates for western blotting (1 × 10^5^ cells/well). In the cell cycle analysis using flow cytometry, cells were fixed with ice-cold 70% ethanol, re-suspended in PBS containing both RNase A (1 mg/ml) and propidium iodide (1 mg/ml), incubated at 37 °C for 30 min, and subjected to flow cytometry (BD Bioscience, Franklin Lakes, NJ, USA), as described previously^36^. The results obtained were analyzed with DIVA software (BD Bioscience). Cell lysates prepared with CelLytic M cell lysis reagent (Sigma-Aldrich, St Louis, MO, USA) were subjected to Western blotting for the detection of PLK1 and β-actin with an anti-PLK1 mouse monoclonal antibody (1:1000; Abcam, Cambridge, UK) and anti-β-actin mouse monoclonal antibody (1:500; BioVision Research Products, Mountain View, CA, USA), respectively. In time-lapse imaging, HeLa-S FUCCI cells were seeded in glass-coated 3.5-cm dishes (5 × 10^4^ cells/dish) and cultured for 12 h. Just after the addition of the indicated concentrations of TAK-960, orange (monomeric Kusabira-Orange2: mKO2) and green fluorescence (monomeric Azami-Green1: mAG1) emitted from HeLa-S FUCCI cells was observed with FLUOVIEW FV10i (Olympus, Tokyo, Japan) every 20 min for 72 h.

### Clonogenic survival assay

Cells (100, 200, 700, and 2,000 HeLa cells/60-mm dish, 200, 300, 500, and 1,000 H1299 cells/60-mm dish, and 100, 200, 1,000, and 10,000 HCT116 cells/60-mm dish for 0, 2, 4, and 6 Gy, respectively) were precultured in medium containing TAK-960 for 0 (Fig. 2d) or 12 h (Figs 2a–c and 3d–f). These cells were then exposed to 0, 2, 4, or 6 Gy X-radiation (ACROBIO Co., Tokyo, Japan) just after replacing the culture medium to a TAK-960-free one. The cells were further cultured for 2 weeks. Surviving colonies were fixed with 70% ethanol and stained with Giemsa solution. Colonies consisting of more than 50 cells were counted as surviving colonies. The plating efficiency and surviving fraction were calculated as described previously^37^.

### Transient transfection

Cells were transiently transfected with pcDNA3.1/myc-His (as an empty vector (EV)), pcDNA3.1/PLK1, pcDNA3.1/PLK1-R136G, pcDNA3.1/PLK1-T210D, or pcDNA3.1/PLK1-R136G&T210D using the Polyfection transfection reagent (Qiagen Inc., Valencia, CA, USA) according to the manufacturer’s instructions. These cells were then cultured for 48 h and subjected to each *in vitro* experiment.

### Tumor-bearing mice, TAK-960 treatment, and radiation treatment *in vivo*

The suspension of HeLa cells (2 × 10^6^ in 100 μL PBS) or H1299 cells (3 × 10^6^ in 100 μL PBS) was subcutaneously inoculated into the right hind legs of 8-week-old nude mice (BALB/c nu/nu mice; SHIMIZU Laboratory Supplies Co., Ltd; Kyoto, Japan). The indicated dose of TAK-960 was orally administered to tumor-bearing mice. In the radiation treatment, tumor xenografts were locally irradiated with the indicated dose of ^137^Cs γ-rays using a Gammacell 40 Exactor (MDS Nordion International Inc., Ottawa, Ontario, Canada).

### Immunohistochemical analyses

HeLa tumor xenografts were surgically excised 24 h after the administration of TAK-960. The tumor xenografts were embedded in OCT compound and frozen at −80 °C. Frozen sections were rinsed quickly in PBS, fixed in 4% paraformaldehyde for 10 min, blocked with the serum-free protein block solution (Dako, Glostrup, Denmark) for 20 min, and permeabilized with 0.2% Triton X-100 in PBS for 10 min. The sections were then treated with an anti-pHH3(S10) mouse monoclonal antibody (1:1000; Abcam, Cambridge, MA, USA). pHH3(S10) was detected with Alexa Fluor 594 goat anti-mouse IgG (1:1000; Invitrogen Corp.). The reproducibility of each staining was confirmed in at least three independent tumors and representative results are shown.

### Immunocytochemical analyses

HeLa-S FUCCI cells on glass coverslips were fixed with ice-cold acetone for 2 min and permeabilized with 0.2% Triton X-100 in PBS for 10 min. After being incubated for 20 min in the serum-free protein block solution, cells were treated with an anti-pHH3(S10)-antibody (1:1000). pHH3(S10) was detected with Alexa Fluor 594 donkey anti-mouse IgG (1:1000; Invitrogen Corp.). The reproducibility of each staining was confirmed in at least three independent tumors and representative results are shown.

### Optical imaging of fluorescence in tumor xenografts with HeLa-S FUCCI cells

Optical *in vivo* imaging was carried out with an IVIS-SPECTRUM (Caliper). Fluorescence was acquired with the filter sets EX/535 nm and EM/580 nm for mKO2 and with EX/465 nm and EM/520 nm for mAG1. During imaging, mice were anesthetized with 2.5% isoflurane gas in the oxygen flow (1.5 L per min). Signal intensities were quantified and analyzed using Living Image 2.50-Igor Pro 4.90 software (Caliper), as described previously^38^,^39^,^40^.

### Growth delay assay

Tumor volumes were calculated as 0.5 × length × width^2^, and compared with the initial value to calculate relative tumor volume.

### Statistical analyses

The significance of differences was determined using the Student’s *t*-test. A *P* value < 0.05 was considered to be significant.

### Ethics of animal experiments

All animal experiments were approved by the Animal Research Committee of Kyoto University, and performed according to guidelines governing animal care in Japan.

## Additional Information

**How to cite this article**: Inoue, M. *et al.* PLK1 blockade enhances therapeutic effects of radiation by inducing cell cycle arrest at the mitotic phase. *Sci. Rep.* **5**, 15666; doi: 10.1038/srep15666 (2015).

## Supplementary Material

Supplementary Figure S1-S6, Tables S1-S2

Supplementary Video S1

Supplementary Video S2

## Figures and Tables

**Table 1:** D_10_ values (The dose of radiation required to reduce the number of surviving colonies by 90%) in clonogenic survival assays of Fig. 2a–c.

Cell line	DMSO + RT (Gy)	TAK-960 + RT (Gy)
HeLa	4.2 ± 0.2	3.4 ± 0.3
H1299	7.5 ± 0.6	5.0 ± 0.6
HCT116	3.7 ± 0.3	3.0 ± 0.2

**Table 2:** Statistical analyses of tumor growth delays in Fig. 5a (HeLa xenograft) and c (H1299 Xenograft).

HeLa	Vehicle	9.1 ± 1.7
	Vehicle + RT	14.0 ± 5.1
	TAK-960	8.3 ± 1.6 (NS vs. Vehicle)
	TAK-960 + RT	27.4 ± 10.0 (*P* < 0.05 vs. Vehicle + RT)
H1299	Vehicle	11.9 ± 2.8
	Vehicle + RT	22.0 ± 5.0
	TAK-960	14.4 ± 4.2 (NS vs. Vehicle)
	TAK-960 + RT	30.4 ± 5.9 (*P* < 0.05 vs. Vehicle + RT)

Results are the means of the days on which the relative tumor volume of each tumor reached two (HeLa) or five (H1299)-fold that of the initial volume on day 0, ±s.d. *n* = 8 (A mouse bearing a H1299 xenograft in the TAK960 + RT group died on day 1 for undefined reasons).

NS = not significant.

**Table 3:** Statistical analyses of tumor growth delays in Fig. 5e (HeLa xenograft).

Vehicle	8.8 ± 2.5
Vehicle + RT	17.3 ± 3.2
TAK-960	9.8 ± 2.6 (NS vs. Vehicle)
TAK-960 + RT	17.3 ± 5.8 (NS vs. Vehicle + RT)

Results are the means of the days on which the relative tumor volume of each tumor reached two-fold that of the initial volume on day 0, ±s.d. *n* = 8.

NS = not significant.
