# Supplementary material for: PLK1 blockade enhances therapeutic effects of radiation by inducing cell cycle arrest at the mitotic phase
Source: Sci Rep. 2015 Oct 27;5:15666. doi: 10.1038/srep15666 (PMC4621528; doi:10.1038/srep15666)
Supplement: Supplementary Figure S1-S6, Tables S1-S2 [file srep15666-s1.pdf]

Supplementary Information

PLK1 blockade enhances therapeutic effects of radiation by inducing cell cycle arrest at the mitotic phase

Authors:

Minoru INOUE[1,2], Michio YOSHIMURA*[1], Minoru KOBAYASHI[1,2], Akiyo MORINIBU[1,2], Satoshi ITASAKA[1], Masahiro HIRAOKA[1], Hiroshi HARADA*[1,2,3,4]

Affiliations:

[1] Department of Radiation Oncology and Image-applied Therapy, Kyoto University Graduate School of Medicine, 54 Shogoin Kawahara-cho, Sakyo-ku, Kyoto 606-8507, Japan.

[2] Group of Radiation and Tumor Biology, Career-Path Promotion Unit for Young Life Scientists, Kyoto University, Yoshida Konoe-cho, Sakyo-ku, Kyoto 606-8501, Japan.

[3] Precursory Research for Embryonic Science and Technology (PRESTO), Japan Science and Technology (JST), 4-1-8 Honcho, Kawaguchi, Saitama 332-0012, Japan.

[4] Hakubi Center, Kyoto University. Yoshida-Honmachi, Sakyo-ku, Kyoto 606-8501, Japan.

Supplementary Figure S1

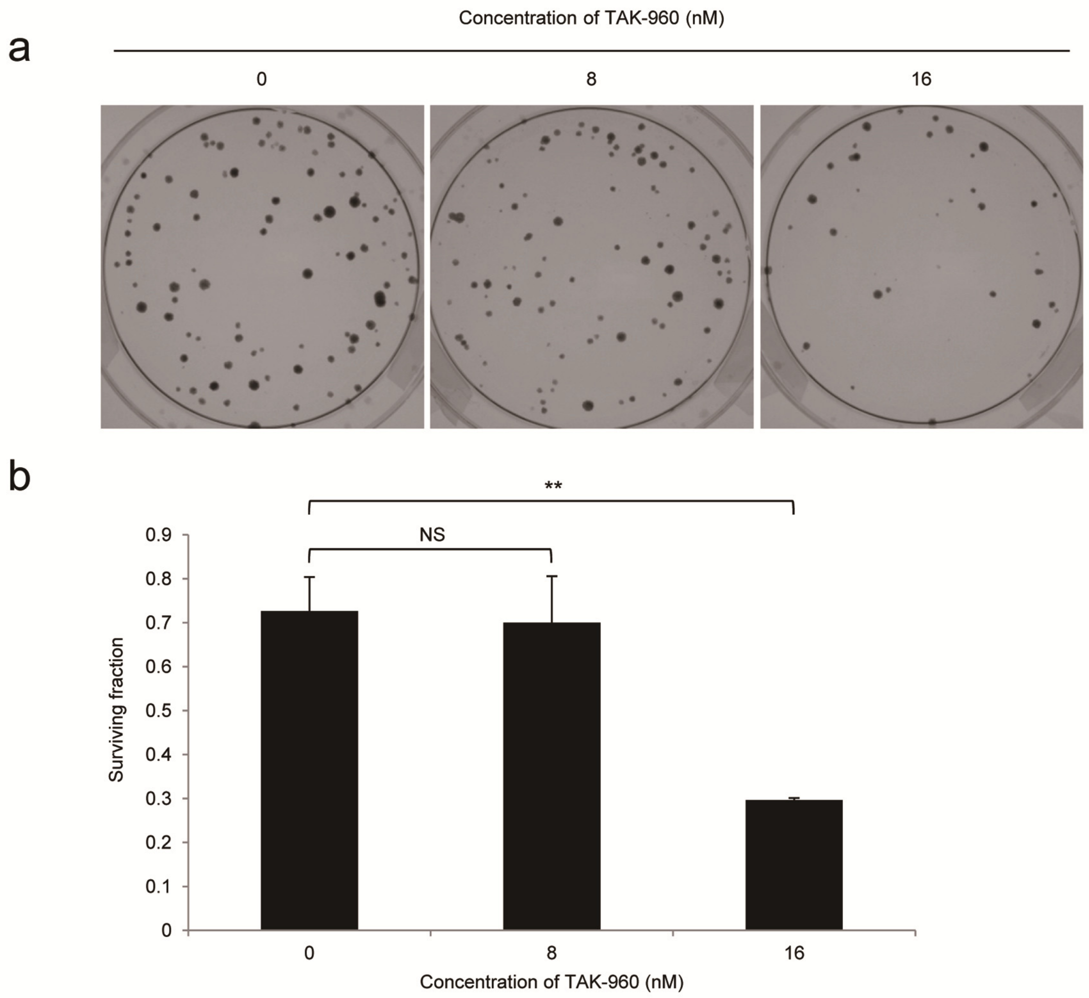

Supplementary Figure S1 | Cytotoxic effects of the TAK-960 treatment *in vitro*. (a, b) HeLa cells were treated with the indicated concentrations of TAK-960 for 12h and subjected to the clonogenic survival assay. (a) Representative images are shown. (b) The surviving fraction is shown. Results are the mean ± s.d. n = 3. **P<0.01. NS = not significant.

Supplementary Figure S2

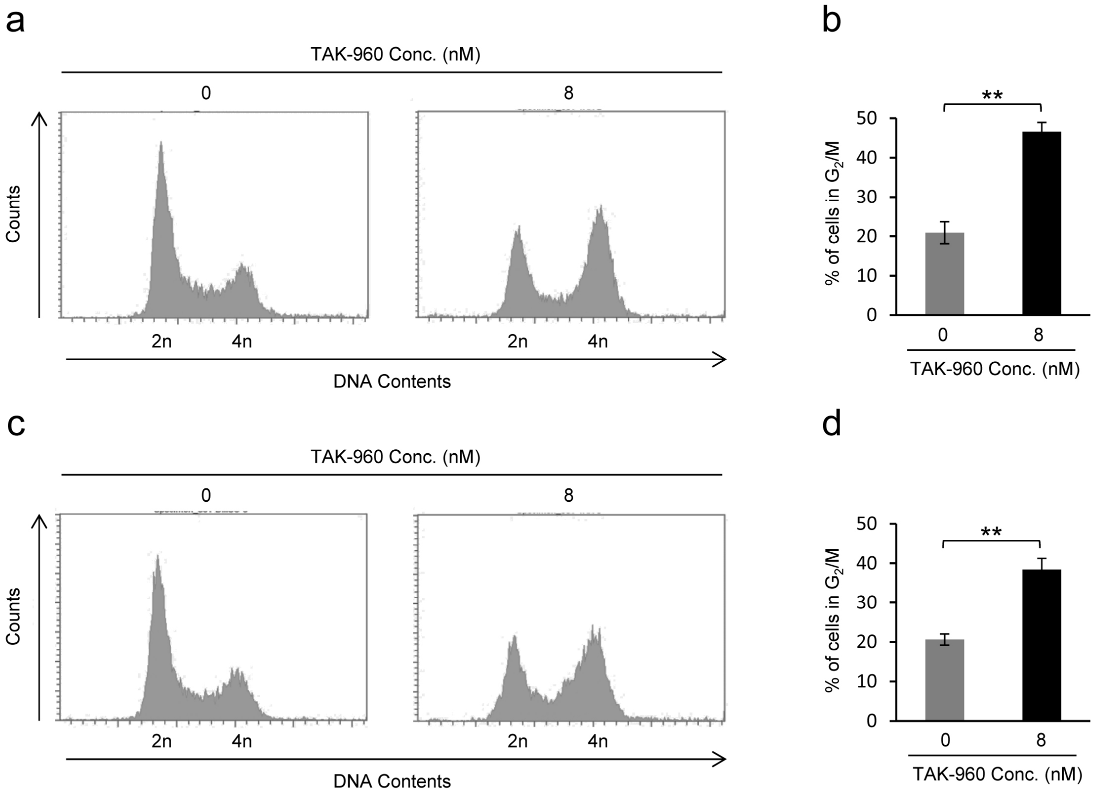

Supplementary Figure S2 | Mitotic arrest of HCT116 and H1299 cells by the TAK-960 treatment *in vitro*. (a-d) HCT116 (a, b) and H1299 (c, d) cells were treated with the indicated concentrations of TAK-960 for 12h and subjected to flow cytometry-based cell cycle analyses. (a, c) Representative data are shown. (b, d) The proportion of HCT116 (b) and H1299 cells in G_2/M phases are quantified using the data in a and c, respectively. Results are the mean ± s.d. n = 3. $^{**}P<0.01$.

Supplementary Figure S3

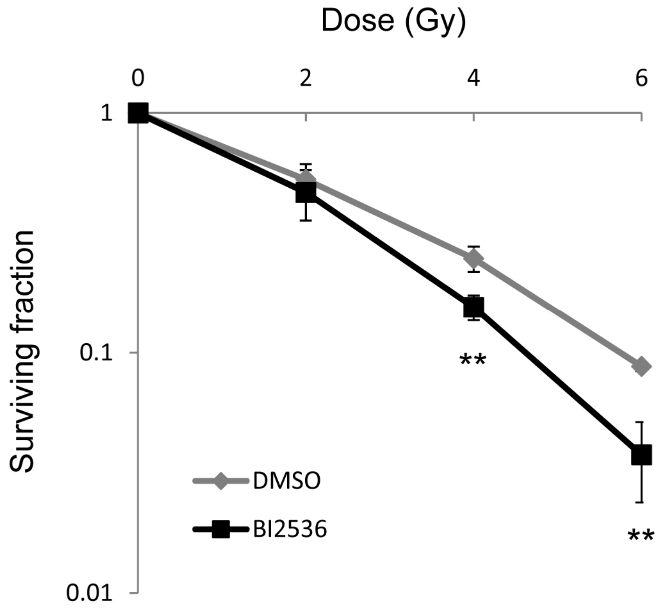

Supplementary Figure S3 | Radiosensitizing effects of BI2536 *in vitro*. A clonogenic survival assay was performed using HeLa cells treated with DMSO or BI2536 for 12 h and with the indicated dose of X-irradiation. A representative graph is shown. Results are the mean ± s.d. *n* = 3. ***P*<0.01.

Supplementary Figure S4

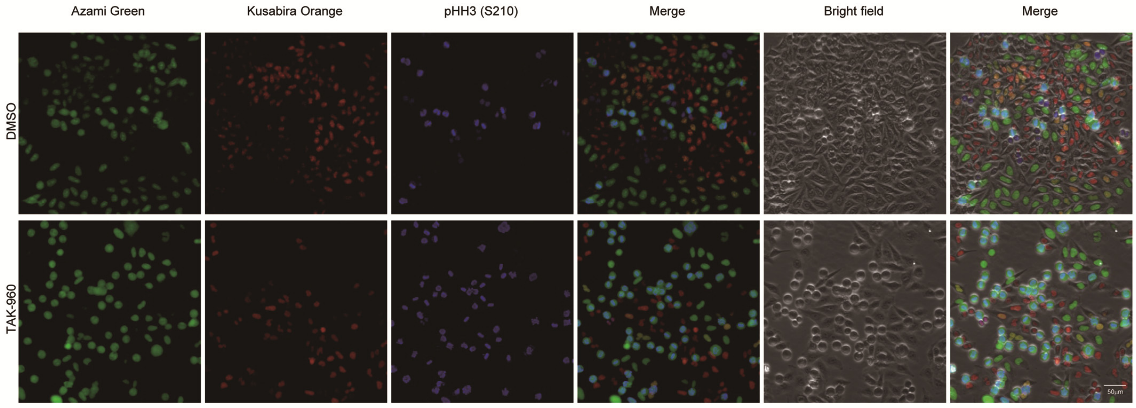

Supplementary Figure S3 | Immunocytochemical analyses of HeLa-S FUCCI cells with pHH3(S10). HeLa-S FUCCI cells were treated with 0 or 8 nM TAK-960 for 12h, briefly fixed with acetone, and subjected to immunocytochemical analyses using an anti-pHH3(S10) mouse monoclonal antibody and Alexa Fluor 633 goat anti-mouse IgG. Bar = 50 μm. Fluorescence from monomeric Azami-Green1 (mAG1), monomeric Kusabira-Orange2 (mKO2), and Alexa Fluor 633 were represented as green, red, and pseudo-blue.

Supplementary Figure S5

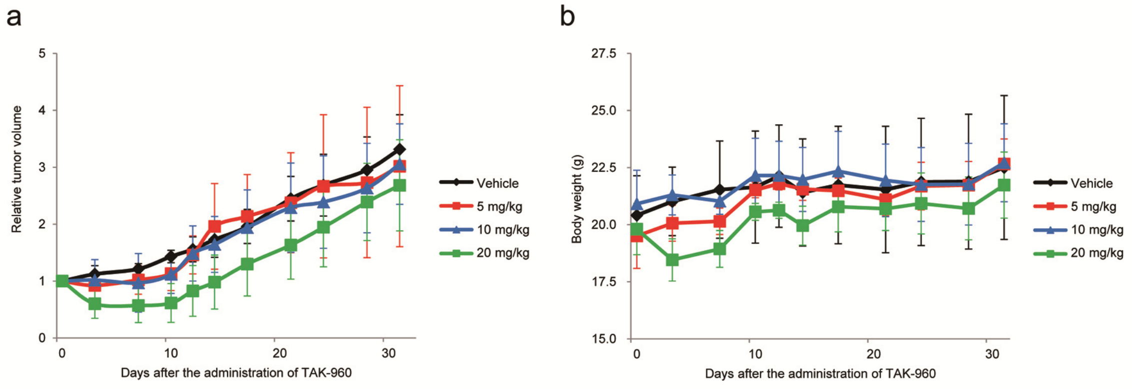

Supplementary Figure S4 | Effects of the TAK-960 treatment on tumor growth. Mice bearing a subcutaneous HeLa-S FUCCI tumor xenograft were administered the indicated dose of TAK-960 on day 0, and subjected to the tumor growth delay assay. (a) Relative tumor volumes are calculated as the ratio of the tumor volume on each day to the corresponding volume on day 0. Results are the means ± s.d. n = 5. (b) Changes in the body weights of mice in the experiment of a. Results are the means ± s.d. n = 5.

Supplementary Figure S6

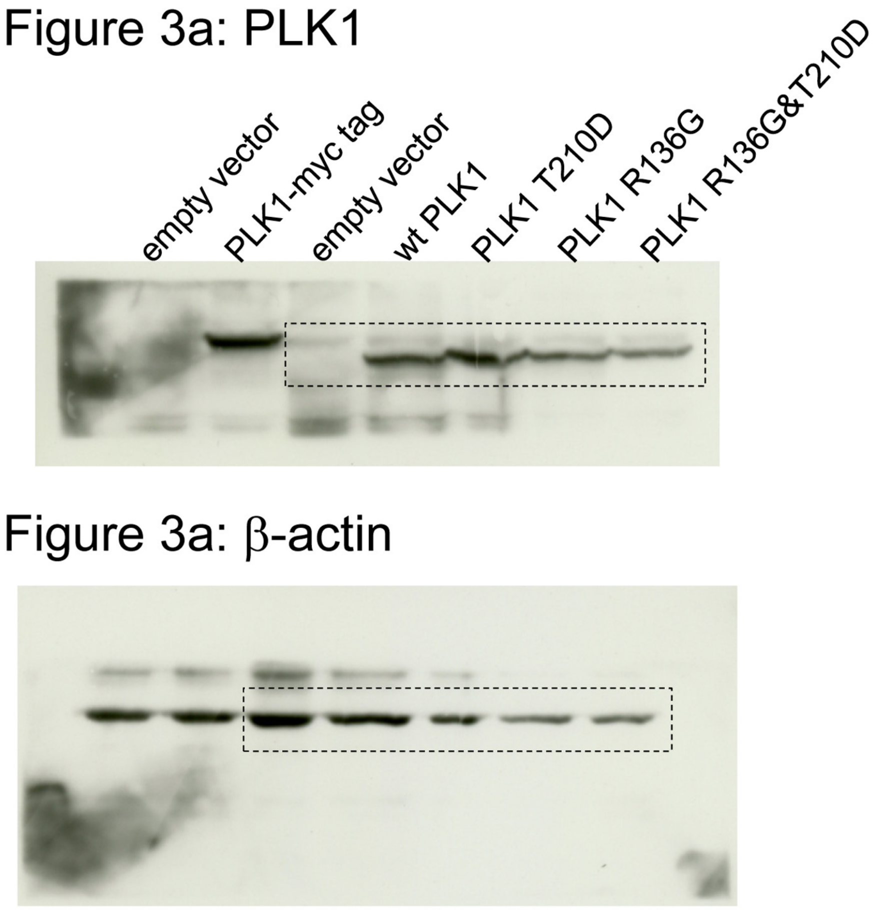

Supplementary Figure S5 | Full blots of the indicated figures.

Supplementary Table S1 | Tumor growth delays at various concentrations of TAK-960

Concentration of TAK-960	TGDT (days ± SD)
Vehicle	20.4 ± 7.8
5 mg/kg	22.3 ± 6.3 (P = 0.65 vs. 0 mg/kg)
10 mg/kg	21.8 ± 3.8 (P = 0.75 vs. 0 mg/kg)
20 mg/kg	28.4 ± 5.2 ($P<0.05$ vs. 0 mg/kg)

Abbreviations: TGDT, tumor growth doubling time, SD, standard deviation

Supplementary Table S2 | Population of mitotic cells with or without the TAK-960 treatment

Concentration of TAK-960	Population of mitotic cells (% ± SD)
0 nM	9.4 ± 2.3
8 nM	38.6 ± 3.7 (*P*<0.01)

Abbreviations: SD, standard deviation

Legend of Supplementary Video S1

Time-lapse imaging of HeLa-S FUCCI cells in the absence of TAK-960 treatment.

Legend of Supplementary Video S2

Time-lapse imaging of HeLa-S FUCCI cells in the presence of 8nM TAK-960 treatment.
